# Lung Point in a Case of Bronchoscopy Lung Volume Reduction: Consider Its Mimics Before Inserting the Tube

**DOI:** 10.24908/pocus.v9i2.17551

**Published:** 2024-11-15

**Authors:** Mohannad Wazirali, Paul M Shaniuk

**Affiliations:** 1 Division of Pulmonary, Critical Care, and Sleep Medicine, University Hospitals Cleveland Mediacal Center, Case Western Reserve University School of Medicine Cleveland, OH USA; 2 Faculty of Medicine, King Abdulaziz University Rabigh SAU; 3 School of Medicine, Case Western Reserve University Cleveland, OH USA; 4 Department of Medicine, Louis Stokes Cleveland Veterans Affairs Medical Center Cleveland, OH USA; 5 Inpatient Medical Services, VA Northeast Ohio Healthcare System Cleveland, OH USA; 6 Medicine and Pediatrics, Case Western Reserve University Cleveland, OH USA; 7 Internal Medicine and Cardiology, VA Northeast Ohio Healthcare System Cleveland, OH USA; 8 Internal Medicine, University Hospitals Cleveland Medical Center Cleveland, OH USA

**Keywords:** Lung Ultrasound, BLVR, Pneumothorax, Lung Point

## Abstract

Point of Care Ultrasound (POCUS) is used to evaluate many clinical scenarios. Chest POCUS has been integrated as a part of a clinical protocol to assess patients with lung pathology 1. The ability to detect pneumothorax using chest POCUS has been shown to be superior to chest radiography, with specificity reported to be as high as 100% when a lung point sign is identified. In addition to improved diagnostic accuracy, chest POCUS has the added benefits of ease of access and absence of ionizing radiation. Here we describe a case where a patient with a high pre-test probability for pneumothorax had a detected lung point sign, but pneumothorax was ruled out via Computed Tomography (CT). This case highlights the importance of considering the mimics of the lung point sign. This case also shows a unique and interesting finding related to pleural movement restriction post-Bronchoscopic lung volume reduction (BLVR).

## Background

Point of Care Ultrasound (POCUS) is gaining popularity and can be used at bedside for immediate answers to clinical questions. One of the bedside applications of POCUS is to diagnose pneumothorax with a pooled sensitivity and specificity of 88% and 99%, respectively. While Computed Tomography (CT) remains the gold standard in diagnosing pneumothorax, chest POCUS has demonstrated superior performance compared to chest radiography 2. Multiple POCUS signs have been described in cases of pneumothorax, including absence of lung sliding, absence of B-lines, presence of lung point, absence of lung pulse and adjacent findings by using M-mode and color Doppler 3. Among these signs, detection of lung point is considered pathognomonic with a specificity of 100% in the absence of parietal emphysema 4. Evaluation of post procedural pneumothorax using POCUS has been described in the literature 5, 6. Many of the thoracic and endobronchial procedures carry a high risk of pneumothorax due to the nature of these procedures. Therefore, performing chest POCUS to routinely screen and re-evaluate patients’ post-endobronchial procedures is a very useful clinical application with very high reported negative and positive predictive values 7. Bronchoscopic lung volume reduction (BLVR) is one of these procedures that carries a very high risk of pneumothorax, ranging between 4.2% and 34.4% 8. Additionally, most of the pneumothoraxes post-BLVR occurred in the first three days 8. The current expert consensus is to recommend hospital admission for at least three nights post BLVR with frequent chest radiographs to evaluate for pneumothorax. These patients typically have a lower cardiopulmonary reserve and thus a lower tolerance for pneumothorax than otherwise healthy individuals 9. Here we describe a case where a lung point was identified in a patient who had chest POCUS performed after he developed chest pain and dyspnea status-post BLVR procedure and thus had a high pre-test probability for pneumothorax. 

## Case Presentation

**Figure 1:**
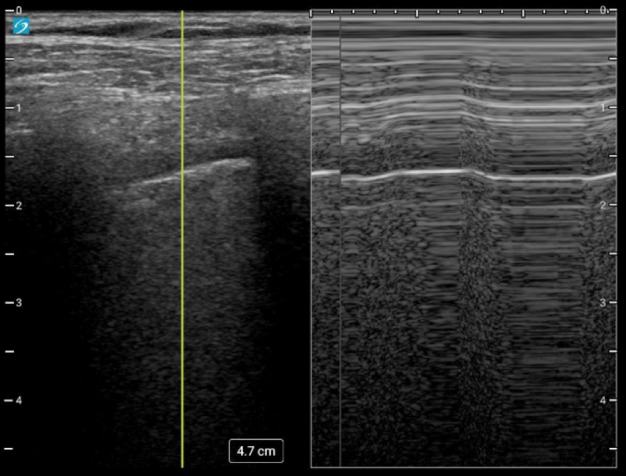
Bar Code Sign Present. M-Mode at the area of the lung point showing the transition from a “seashore sign” to a “bar code sign”

**Figure 2:**
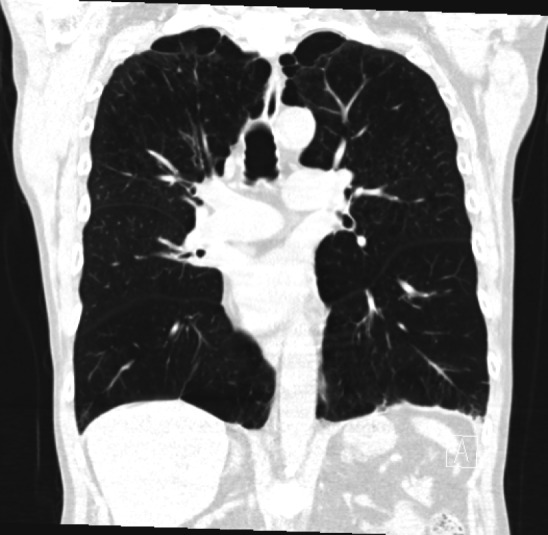
Coronal computed tomography image of the chest showingbiapical subpleural blebs but no identifiable pneumothorax.

**Figure 3:**
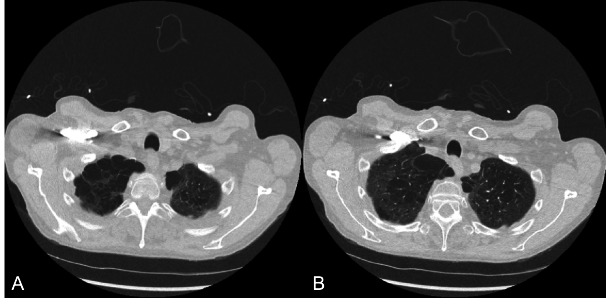
And B: Axial computed tomography images of the chest at the level of the apical subpleural blebs in the left side.

Our patient was a 68-year-old male with a medical history of very severe Chronic Obstructive Pulmonary Disease (COPD) with emphysema and hyperinflation who was admitted electively to the hospital for BLVR using Zephyr ® valves. Most recent pulmonary function testing prior to the procedure showed forced vital capacity (FVC) of 3.63L (78% of predicted), forced respiratory volume (FEV)1 of 0.92L (26% of predicted), FEV1/FVC of 25.3%, total lung capacity (TLC) of 9.64L (123% of predicted), residual volume (RV) of 6.05L (231% of predicted) and diffusion capacity for carbon monoxide (DLCO) of 8.9 ml/min/mmHg (31% of predicted). CT evaluation of the chest prior to the procedure showed severe homogenous emphysema involving all the lung lobes. A total of five valves were placed successfully in the left upper lobe and he was transferred to the intensive care unit for close monitoring per the hospital protocol for BLVR. On his second post procedural day, he started to have chest discomfort and dyspnea at rest that progressed on the second day with unremarkable work up including chest radiograph. An educational POCUS exam was performed as part of an ultrasound elective clinical rotation, and the patient was found to have absent pleural sliding over the left upper anterior chest wall at the mid-clavicular line (Video S1 and Video S2) with no B-lines and bar code sign (Figure 1) with detectable lung point (Video S3). The findings were relayed to the treating physicians who ordered a confirmatory chest CT, and the patient was sent for CT (Figures 2 and 3). This was negative for pneumothorax but revealed a small area of sub pleural bullous emphysema at the lung apex. However, this area was medial to the mid-clavicular line where the abnormal lung signs were identified with POCUS.

The accuracy of diagnosing pneumothorax can vary based on the detected signs during the examination. While absence of lung sliding has a sensitivity for pneumothorax of 100% and a specificity of 78%, identifying a lung point has a lower sensitivity of 79% but a reported specificity of 100% in the absence of parietal emphysema (4). Multiple reasons for false negative results have been described such as very small pneumothorax, unevenly distributed pneumothorax, and mistaking the cardiac effect on the left lung as lung sliding or subcutaneous emphysema. Other conditions with limited lung movement and absence of lung sliding – as in severe COPD, ventilation with high positive end expiratory pressure, right main stem intubation, bullous emphysema, pleural adhesions acute respiratory distress syndrome or lung contusion – were described with false positive results 10. Few mimics of the lung point sign should make the clinician more conscious while interpreting this “virtually pathognomonic” sign which adds more limitations on top of its limited sensitivity 2, 11, 12. Cases of lung point mimics have been reported 13. One of these situations is the physiological lung point where the visceral pleura comes in contact with the mediastinal pleura creating a lung point. However, the A-Profile is absent in the no-lung area and cardiac pulse can be seen in the physiological lung point which differentiate it from a true lung point 14. Another situation is in cases of pulmonary contusion where the contused lung interfaces with the normal lung, creating a pseudo-lung point due to the difference in the lung densities but with absent barcode sign (also referred to as stratosphere sign) 15. Similar concept to the pseudo-lung point described in trauma cases is seen in infants with transient tachypnea of the newborn (TTN), where relatively normal lung area interfaces with area of pulmonary edema. It is referred to as the double lung point sign and it is diagnostic for TTN 1. Lastly, the Bleb point sign in cases of severe bullous emphysema has been identified as a lung point mimic, although it was demonstrated that ultrasound can differentiate between pneumothorax and bullous emphysema 16. However, the bleb point sign requires complete absence of lung tissue between the pleura and the bulla and in this case, it will be identical to the lung point sign and can only be distinguished by using CT 17. This essentially makes it a true false positive lung point 17. 

In our case, we describe a patient with high pre-test probability for pneumothorax due to his recent BLVR procedure, which carries a high risk of pneumothorax. Multiple signs of pneumothorax were detected on chest POCUS, including the most specific sign (the lung point) and possible mimics of this sign were excluded. However, confirmatory chest CT was negative for pneumothorax. To our knowledge, this finding was not described in post-BLVR cases. However, the concept of detectable lung point due to pleural movement restriction is not fundamentally new. Steenvoorden et al. reported a case of detected lung point in a patient with asbestos-related pleural disease that resulted in pleural thickening and restricted movement 18. In our case, we hypothesize that the absent lung sliding and detection of lung point could be explained by the presence of the endobronchial valves leading to restricted pleural movement in a similar situation to the case reported by Steenvoorden et al. There is a possibility that the area with sub-pleural emphysema detected at the lung apex by CT scan led to false positive signs of pneumothorax by our POCUS examination. However, we believe that area was medial to the area we scanned. Our case adds to the sparse evidence about the limitations of the lung point sign in detecting pneumothorax. Further evaluation with other imaging modalities in non-emergency situations might be needed before deciding on an intervention based solely on the POCUS findings.

## Informed Consent Statement 

A written informed consent was obtained from the patient representative that included a description of the case report, the information and images being used, the risks and benefits and the voluntary nature of the participation.

## Disclosure Statement 

The authors declare that they have no competing interests.

## Supplementary Material

 Video S1

Video S2

Video S3
